# Copper, Manganese, Zinc, and Cadmium in Tea Leaves of Different Types and Origin

**DOI:** 10.1007/s12011-017-1140-x

**Published:** 2017-09-02

**Authors:** W. Podwika, K. Kleszcz, M. Krośniak, P. Zagrodzki

**Affiliations:** 1 State Higher Vocational School in Tarnow, Mickiewicza 8 St., 33-100 Tarnow, Poland; 2 Department of Food Chemistry and Nutrition, Medical College, Jagiellonian University, Medyczna 9 St., 30-688 Cracow, Poland

**Keywords:** Tea, Cadmium, Zinc, Manganese, Copper, AAS

## Abstract

Concentrations of selected metals (Cu, Mn, Zn, Cd) in tea leaves were investigated. Samples included black, green, and other (red, white, yellow, and oolong) teas. They were purchased on a local market but they covered different countries of origin. Beverages like yerba mate, rooibos, and fruit teas were also included in the discussion. Metal determinations were performed using atomic absorption spectrometry. In black teas, Mn/Cd ratio was found to be significantly higher (48,091 ± 35,436) vs. green (21,319 ± 16,396) or other teas (15,692 ± 8393), while Cd concentration was lower (31.4 ± 18.3 μg/kg) vs. other teas 67.0 (67.0 ± 24.4). Moreover, Zn/Cu and Cu/Cd ratios were, respectively, lower (1.1 ± 0.2 vs. 2.2 ± 0.5) and higher (1086 ± 978 vs. 261 ± 128) when comparing black teas with other teas. Intake of each metal from drinking tea was estimated based on the extraction levels reported by other authors. Contributions to recommended daily intake for Cu, Mn, and Zn were estimated based on the recommendations of international authorities. Except for manganese, tea is not a major dietary source of the studied elements. From the total number of 27 samples, three have shown exceeded cadmium level, according to local regulations.

## Introduction

Tea is an infusion prepared from *Camellia sinensis* leaves originating from China and some parts of India. Nowadays, this evergreen shrub is grown in many other countries, such as Japan, Indonesia, Sri Lanka, Kenya, and Vietnam. The conditions of growing tea shrub, e.g., location, climate, and soil, have an impact on the taste and properties of the final beverage [1].

Tea is the one of the most popular beverages in the world. Six types of tea are distinguished as follows: green, white, yellow, oolong (blue tea/semi-fermented tea), red, and black. These types differ in terms of oxidation level, color of leaves, and infusion. The oxidation level of tea determines the color of leaves and infusion and it depends on the way the fermentation was carried out. The most popular types of tea are the following: green tea, which preserves the properties of fresh leaves to the highest degree, and black tea, which is obtained after full fermentation of fresh leaves.

Among other components, tea leaves consist of tanning agents, alkaloids, amino acids, pigments, and trace amounts of mineral compounds. Trace elements present in tea leaves play an important role in human metabolism [2]. They can be released from leaves to the infusion. Thus, tea leaves may become the source of metals in human diet. Some of the metallic elements, such as copper, manganese, and zinc are essential for basic processes in the human body while others (like cadmium) are toxic. The main aim of our study was to determine the content of copper, manganese, zinc, and cadmium in tea leaves and to find out if there are any significant differences among the teas of different types and countries of their origin.

## Materials and Methods

### Samples

Tea samples (*n* = 27) were purchased on a local market in southern Poland. All samples were based on pure tea leaves, without additives or flavors. The set of samples included teas in the form of loose leaves as well as ground ones in tea bags. Samples were divided according to their type and country of origin. Among the samples, there were 12 samples of black tea, eight samples of green tea, two samples of red tea, two samples of white tea, two samples of oolong tea, and one sample of yellow tea. All details are given in Table 1.

**Table 1:** Details of samples’ characteristics

Sample no.	Type of tea	Form	Country of origin	Brand name
1	Green	Loose leaves	China	Dynasty
2			Sri Lanka	Sencha
3			India	Assam OP
4			Indonesia	Green Hills, Gunpowder Pearl
5			Japan	Sencha
6		Tea bags	China	Oscar, Green Tea Bags, Yunnan
7			Sri Lanka	Dilmah, Pure Green
8			Vietnam/China	Packers Best
9	Black	Loose leaves	China	Golden Dynasty
10			Sri Lanka	Dimbula Uduwela
11			India	Hattialli
12			Vietnam	Vietnam
13			Kenya	Green Hills, Kenya Pearl
14			No data	Lipton
15		Tea bags	Sri Lanka	Jones Classic
16			Kenya	Loyd, Black Sense, Kenya
17			Argentina, Iran, Indonesia (mix)	Ulubiona
18			No data	Assuan
19			No data	Assam, Teekane
20			No data	Minutka
21	White	Loose leaves	China	Pai Mu Tan
22			China	Shou Mei
23	Red	Loose leaves	China	Pu-erh
24		Tea bags	China	Pu-erh
25	oolong	Loose leaves	Taiwan	Formosa
26			China	Tie Guan Yin Wangjia
27	Yellow	Loose leaves	China	Yellow Tea

## Methods

Prior to the measurements, samples were dried for 2 h at 105 °C. Moisture content (calculated from the mass difference prior to and after drying) varied between 3.5% and 8.4%. From each sample, approximately 0.5 g was taken for the analysis, and microwave-assisted wet digestion with ultrapure nitric acid was carried out. Certified Reference Material (CRM No. 7, Tea Leaves, National Institute of Environmental Studies) was included in the analysis (two samples) in order to provide quality assurance.

All determinations were performed using a Perkin-Elmer 5100 ZL atomic absorption spectrometer. Cadmium was determined using electrothermal atomization, while the rest of the elements were determined using flame technique.

### Statistical Approach

For samples divided into three groups, black teas, green teas, and the others, the descriptive statistics were calculated. The comparisons between groups were performed using the Kruskal-Wallis test with the Dunn post hoc test. Statistical calculations were carried out using commercially available packages Statistica v. 12.5 (StatSoft, Tulsa, USA) and GraphPad InStat v. 3.05 (GraphPad Software, La Jolla, USA).

## Results and Discussion

The results for CRM are listed in Table 2 and for tea samples in Table 3. Descriptive statistics for black, green, and other teas are given in Table 4.

**Table 2:** Results for Certified Reference Materials

CRM	Element	Measured value [mg/kg]	Certified value [mg/kg]
Sample 1	Mn	701 ± 15	700 ± 25
Sample 2		716 ± 8	
Sample 1	Zn	32.3 ± 0.2	33 ± 3
Sample 2		31.2 ± 0.7	
Sample 1	Cd	0.033 ± 0.001	0.030 ± 0.003
Sample 2		0.030 ± 0.001	
Sample 1	Cu	6.8 ± 0.2	7.0 ± 0.3
Sample 2		6.9 ± 0.4

**Table 3:** Concentration of the studied metals in tea samples

Sample no.	Cu [mg/kg]	RSD [%]	Mn [mg/kg]	RSD [%]	Zn [mg/kg]	RSD [%]	Cd [μg/kg]	RSD [%]
1	16.7	1.0	593	1.8	21.3	5.2	37	14
2	15.1	2.2	746	1.2	25.2	0.2	64.3	1.6
3	18.0	1.8	607	1.1	25.2	0.5	44.4	2.3
4	26.6	1.8	457	0.80	18.8	1.2	14	14
5	12.1	4.3	900	2.4	12.6	1.4	15.7	1.6
6	22.6	0.4	582	1.6	28.7	1.8	28.2	4.3
7	16.8	2.2	1590	1.7	23.4	0.7	129	0.07
8	11.8	0.5	1035	1.5	17.8	2.4	153	5.3
9	25.0	1.8	1080	2.8	29.1	0.7	34.7	1.9
10	19.1	0.6	717	0.42	13.3	0.5	6.0	13
11	32.7	1.2	626	2.2	26.5	0.5	26.3	3.6
12	17.3	0.1	1180	1.7	21.1	2.4	42.0	0.05
13	31.0	1.2	2210	1.6	25.9	0.8	64.3	7.0
14	22.5	1.8	831	1.9	27.0	1.7	35.6	1.5
15	30.9	1.0	561	4.1	26.3	0.2	13.0	12
16	14.1	6.0	1020	2.0	20.0	1.8	34.5	8.8
17	16.4	5.5	1290	2.6	17.9	4.1	59.9	3.5
18	16.7	4.5	1620	2.3	14.7	5.8	28.8	5.6
19	14.9	4.5	876	2.5	20.4	0.7	7.9	5.6
20	15.2	2.1	1130	2.0	19.9	1.7	24.0	0.14
21	15.4	1.3	1036	1.1	34.2	0.3	47.3	7.1
22	15.6	7.0	1100	2.5	25.0	2.5	35.6	9.1
23	22.7	2.1	990	2.8	31.8	3.7	58.5	5.8
24	21.7	4.7	730	3.1	45.5	0.3	106	0.51
25	9.2	5.3	949	3.4	22.0	0.6	89.7	1.9
26	9.1	1.7	986	1.5	23.4	0.8	71.6	2.6
27	14.6	7.6	541	1.1	42.4	0.8	60.4	1.2

**Table 4:** Descriptive statistics for studied parameters in the samples; mean values with the same letter in upper index differ significantly

Parameter	*N*	Mean	Median	Min	Max	SD	Kurtosis
Black teas							
Mn (mg/kg)	12	1094.1	1047.9	561.2	2209.7	460.5	2.210
Zn (mg/kg)	12	21.8	20.8	13.3	29.1	5.1	− 1.061
Cd (μg/kg)	12	31.4^a^	31.7	5.5	64.3	18.3	− 0.223
Cu (mg/kg)	12	21.3	18.2	14.1	32.7	6.9	− 1.203
Mn/Zn	12	53.3	52.3	21.3	109.7	25.9	0.691
Mn/Cd	12	48091^b,c^	32747	21532	129907	35436	2.116
Mn/Cu	12	56.2	63.5	18.1	96.8	24.9	− 0.950
Zn/Cd	12	1095	794	298	3022	882	0.758
Zn/Cu	12	1.1^d^	1.1	0.7	1.4	0.2	− 1.546
Cu/Cd	12	1086^e^	633	274	3453	978	2.046
Green teas							
Mn (mg/kg)	8	814.3	676.5	457	1593.6	366.7	2.587
Zn (mg/kg)	8	21.6	22.3	12.6	28.7	5.1	0.021
Cd (μg/kg)	8	60.7	40.8	14.1	153	52.4	− 0.166
Cu (mg/kg)	8	17.5	16.8	11.8	26.6	5.0	0.183
Mn/Zn	8	40.5	28.8	20.3	71.3	21.5	− 1.807
Mn/Cd	8	21319^b^	14821	6767	57174	16396	3.318
Mn/Cu	8	52.3	42.5	17.2	94.6	29.6	− 1.641
Zn/Cd	8	650	567	116	1335	435	− 1.165
Zn/Cu	8	1.3	1.3	0.7	1.7	0.3	1.235
Cu/Cd	8	594	428	77	1885	587	3.557
Other teas							
Mn (mg/kg)	7	903.9	985.6	541.2	1096.7	196.9	0.815
Zn (mg/kg)	7	32.1	31.8	22.0	45.5	9.3	− 1.476
Cd (μg/kg)	7	67.0^a^	60.4	35.6	106	24.4	− 0.427
Cu (mg/kg)	7	15.5	15.4	9.1	22.7	5.4	− 1.166
Mn/Zn	7	31.3	31.2	12.8	43.8	12.8	− 1.449
Mn/Cd	7	15692^c^	13768	6865	30836	8393	0.517
Mn/Cu	7	66.2	67.2	33.6	108.2	30.5	− 1.563
Zn/Cd	7	525	543	246	723	196	− 1.815
Zn/Cu	7	2.2^d^	2.2	1.4	2.9	0.5	− 0.737
Cu/Cd	7	261^e^	242	103	438	128	− 1.543

^a^*p* < 0.05; ^b^*p* < 0.05; ^c^*p* < 0.01; ^d^*p* < 0.001; ^e^*p* < 0.01

### Copper

Copper concentration in the samples varied from 9.1 ± 0.2 to 32.7 ± 0.4 mg/kg (mean 18.7 ± 6.3 mg/kg). Street et al. [1] reported similar, though slightly higher values, between 9 and 65 mg/kg (for the group of 30 samples). Similar results were obtained by Ashraf and Mian [3] as well as by Narin et al. [4]. In our study, the mean concentration of copper in black teas was determined to be 21.3 ± 6.9 mg/kg and for green teas 17.5 ± 5.0 mg/kg. These values were somewhat higher than those reported by Srividhya et al. [2] (14.34 ± 0.49 mg/kg for black teas and 11.28 ± 0.08 mg/kg for green ones, respectively) but, at the same time, lower than reported by Gajewska et al. [5] (black teas 31.3 ± 11.2 mg/kg; green teas 20.0 ± 5.9 mg/kg). The common point is, however, that both Srividhya et al. [2] and Gajewska et al. [5] found higher copper concentrations in black teas than in the green ones, which were also noticed in the present work. Among all types, the highest levels of copper were found in the red and black teas. Copper concentrations in white (15.5 ± 0.1 mg/kg) and oolong (9.15 ± 0.07 mg/kg) teas found in our study are similar to the results of other researchers (Marcos et al. [6], McKenzie et al. [7], Xie et al. [8]), although higher results also have been reported (Malik et al. [9] 17.6–31.6 mg/kg for white and 15.1–25.8 mg/kg for oolong tea). Copper content in red teas (22.0 ± 0.7 mg/kg) is comparable with the results of McKenzie et al. [7] (range 15–43 mg/kg).

### Manganese

Manganese concentration in the samples varied from 457 ± 4 to 2210 ± 35 mg/kg (mean ± SD 962 ± 388 mg/kg). Similar results were published by Street et al. [1], where manganese concentration in 30 samples of different types of teas varied from 511 to 2220 mg/kg. The authors did not notice a major difference between manganese concentration in black and green teas (nor they did for other elements: iron, zinc, and copper).

According to the type of the tea, the highest average concentration of manganese was observed for black teas (1094 ± 460 mg/kg). This value is similar to the results from other studies [2, 5] which also have shown higher manganese concentration in black teas as compared to the green ones. According to Ashraf and Mian, Mn concentration in black tea samples studied by them was within the range of 448–1072 mg/kg [3], while in the study of Narin et al., this range was 564–1082 mg/kg [4]. Both these studies provided results with lower maximum values than the ones observed in our study. Interestingly, tea leaves from Kenya contained apparently the highest concentration of manganese (maximum of 2210 mg/kg) among all countries of the samples’ origin. However, since only two samples from Kenya were analyzed, one should avoid making definite conclusions. Manganese concentration in white (1068 ± 45 mg/kg) and oolong (968 ± 26 mg/kg) teas was similar to reported elsewhere (McKenzie et al. [7], Xie et al. [8]), although relatively low results can be found (Malik et al. [9] 293–479 mg/kg for white tea, which is, in general, low concentration, regardless of the type of tea). Manganese content in red teas (860 ± 183 mg/kg) is comparable with the results of McKenzie et al. (range 615–1268 mg/kg) [7].

### Zinc

Zinc concentration in all samples varied between 12.6 ± 0.2 and 45.5 ± 0.1 mg/kg. The mean value equaled 24.4 ± 7.7 mg/kg and was in line with the results reported by Srividhya et al., 25.39 ± 0.59 mg/kg for black teas and 26.39 ± 0.92 mg/kg for the green ones [2]. Results published by Street et al. were higher (21.5–75.2 mg/kg) than in our study [1].

Our results of zinc concentration in green and black teas are similar to those reported by Srividhya et al. [2] as well as Gajewska et al. [5] and Narin et al. [4]. On the other hand, the mean value of zinc concentration in black teas given by Ashraf and Mian (65.7 ± 31.3 mg/kg) was much higher than the one found by us, which was (21.8 ± 5.1 mg/kg) [3]. In regard to white and oolong teas, zinc concentration found in our study (29.6 ± 6.5 mg/kg for white tea and 22.7 ± 1.0 mg/kg for oolong tea) are in line with other reports (McKenzie et al. [7], Xie et al. [8], Malik et al. [9]). Zinc content in red teas (38.7 ± 7.7 mg/kg) is comparable with the results of McKenzie et al. (range 26–52 mg/kg) [7].

The highest concentrations of zinc were found in red and yellow tea samples. When referring to the country of origin, the teas from China had the highest zinc concentration while tea samples from other countries (except for Japan) had similar levels of this element.

### Cadmium

Average cadmium concentration in all tea samples equaled 49 ± 36 μg/kg. However, concentration of this element was rather disparate among all studied samples. The highest Cd concentration (153 ± 8 μg/kg) was found in sample no. 8—green tea (tea bag), while the lowest one (6.0 ± 0.8 μg/kg) in sample no. 10—black tea (leaves). These results are much lower than results shown by Ashraf and Mian [3] and Narin et al. [4] who found Cd concentration in black teas to be equal 1.1 ± 0.5 and 2.3 ± 0.4 mg/kg, respectively. Regarding oolong tea, our result (80.6 ± 12.8 μg/kg) is very similar to the one presented by Marcos et al. (82 ± 9 μg/kg; one sample only) [6].

Studies published by Gajewska et al. showed that an average Cd concentration was much higher for black teas (426 ± 506 μg/kg) than for the green ones (218 ± 43 μg/kg) [5]. Contrary to that, our results were in the opposite order; an average Cd concentration was 31.3 ± 18.4 μg/kg for black teas and 60.3 ± 52.8 μg/kg for the green ones. Therefore, not only was the tendency reversed, but also the values apparently differed. However, more recently, Srividhya et al. reported Cd concentration in green teas to be twice as high as for the black teas (1.59 ± 0.26 and 0.89 ± 0.10 mg/kg, respectively), though this result is based on two samples only [2].

Among the tea samples studied by us, the lowest concentration of cadmium was found in black teas and the highest in red and oolong teas. When considering the country of origin, low Cd concentrations were found in teas from Japan and Indonesia while in the sample from Taiwan, it was relatively high. Table 5 shows comparison of our results with other researches.

**Table 5:** Comparison of results of this work with reported by other authors

		This work	Street et al. (2006)^a^	Narin et al. (2004)	Ashraf and Mian (2008)	Srividhya et al. (2011)	Gajewska et al. (2000)
Green tea (mean ± SD)	Number of samples	8	13	–	–	1	14
	Mn [mg/kg]	814 ± 367	1092 ± 440	–	–	508 ± 44	663 ± 168
	Cu [mg/kg]	17.5 ± 5.0	23.6 ± 5.8	–	–	11.28 ± 0.08	20.0 ± 5.9
	Zn [mg/kg]	21.6 ± 5.1	47.2 ± 15.8	–	–	26.39 ± 0.92	45.7 ± 16.1
	Cd [μg/kg]	60.3 ± 52.8	–	–	–	1.59 ± 0.26	218 ± 43^b^
Black tea (mean ± SD)	Number of samples	12	13	14	17	1	6
	Mn [mg/kg]	1094 ± 460	972 ± 448	788 ± 152	751 ± 185	709 ± 14	718 ± 232
	Cu [mg/kg]	21.3 ± 6.9	29.9 ± 13.0	16.5 ± 3.9	18.1 ± 6.9	14.34 ± 0.49	31.3 ± 11.2
	Zn [mg/kg]	21.8 ± 5.1	44.6 ± 12.9	129 ± 13	65.7 ± 31.3	25.39 ± 0.59	52.6 ± 16.8
	Cd [μg/kg]	31.3 ± 18.4	–	2.3 ± 0.4	1.1 ± 0.5	0.89 ± 0.10	426 ± 506^b^

^a^In this publication, results were given for each sample individually; means and SD were calculated by the authors of this work for the purpose of comparison

^b^For Cd, the number of samples was 20

Comparing black and green teas, Mn/Cd ratio was found to be significantly different between these two groups. When comparing black teas to the others, four parameters showed significant differences: Cd concentrations, Mn/Cd, Zn/Cu, and Cu/Cd ratios. Further studies, including more tea samples, are needed to establish if there is such a general trend for these groups of teas.

Since black tea undergoes full fermentation during production, it seems that this process can affect the content of the metals (e.g., removing the metals by rinsing them out from the leaves; the degree of this removal can vary for each metal). Besides this, the content of the given metal in tea leaves obviously depends on its content in the tea plantation soil.

### Daily Intake Estimation

Daily intake of all determined metals with consumed tea infusion for black and green teas was estimated. It was based on the assumptions that the daily consumption of tea is three cups and each one is prepared using 1.5 g of tea leaves. Extraction degrees for each element were taken from the report of Gajewska et al. [5]. Recommended daily intake for Mn, Zn, and Cu was taken from the Dietary Reference Intakes [10]. In addition, the European Union Population Reference Intake for adults is given for comparison [11].

Estimated daily intakes together with the recommendations mentioned above for black and green tea are listed in Table 6. It can be noticed that tea is a major source of manganese in the diet, while intake of other elements is negligible.

**Table 6:** Estimated intakes of the elements from tea compared to recommendations

Element	Extraction degree [%]	Calculated daily intake [mg/day]	Reference daily intake [mg/day] [10]		Population reference intake [mg/day] [11]	
			Male	Female	Male	Female
Black tea						
Mn	28.9	1.42	2.3	1.8	1–10	1–10
Zn	32.5	0.032	11	8	9.5	7
Cu	22.7	0.022	0.9	0.9	1.1	1.1
Green tea						
Mn	30.6	1.12	2.3	1.8	1–10	1–10
Zn	36.1	0.035	11	8	9.5	7
Cu	22.6	0.017	0.9	0.9	1.1	1.1

Since cadmium is considered a toxic element, there is no RDI defined for it. Cadmium content in the samples can be compared with the upper allowed level of this element in tea, which was established by ordinance of the Health Minister of Poland [12] and equals 0.10 mg/kg of dry material. It can be noticed from Table 3 that only in three samples (denoted 7, 8, and 24) was this level exceeded.

### Comparison with Other Types of Teas

In separate studies, other types of tea, and similar beverages, were examined. Schunk et al. [13] analyzed Brazilian herbal teas (chamomile, lemongrass, fennel, and yerba mate). In that report, the copper concentration varied from 0.19 ± 0.05 to 0.43 ± 0.07 μg/g while the levels of cadmium were much lower—between 0.03 ± 0.01 and 0.05 ± 0.01 μg/g. The highest Mn concentration was found to be 53.45 ± 7.07 μg/g. Results for copper and cadmium overlapped with those obtained in our work, but the content of manganese was much lower.

Rusinek-Prystupa et al. [14] examined teas containing yerba mate and rooibos. For both these groups, the concentration of Zn varied from 7.19 to 106 mg/kg and concentration of Cu was in a range from 1.98 to 14.05 mg/kg (being much lower for rooibos than for yerba mate). These values are in line with our results, with copper content being somewhat lower in rooibos than in ordinary tea. Manganese in yerba mate was present in the concentration from 269 to 2261 mg/kg which is again similar to the results of this work for green and black tea. However, the values found in rooibos were much lower (1.980–3.363 mg/kg).

Brzezicha-Cirocka et al. [15] analyzed eight types of fruit teas. Among all these groups, the concentrations were in a range of 15–30 mg/kg for Cu, 130–770 mg/kg for Mn, and 3.8–15 mg/kg for Zn. These values are, in general, comparable with those found by us in green or black teas (with our results being slightly higher).

### Limitation of the Study

Currently, a large selection of white and red teas is available on the Polish market. Their lower consumption is implicated not only by their relatively higher prices but also by the nutritional habits of Polish consumers. On the other hand, it is well known that white teas have rich chemical composition (including the highest polyphenol content among all teas and therefore strong antioxidant activity). Similarly, it is believed that red teas also exert a variety of biological functions. For this reason, it is important to examine such teas, paying special attention to trace elements as well as other ingredients with favorable properties in human diet—in order to convince consumers to drink them more often. Small number of such teas examined in our study is its limitation and deserves next research particularly focused on white and red teas as possible candidates for potent functional food.

## Conclusions

In black teas, Mn/Cd ratio was found to be significantly higher vs. green or other teas, while Cd concentration was lower vs. other teas. Moreover, Zn/Cu and Cu/Cd ratios were, respectively, lower and higher when comparing black teas with other teas. This differentiation can be caused by the fermentation process during black tea production. Our results partly agree with the reports of other researchers; however, some differences can be noticed. In particular, zinc content in black tea as well as cadmium content in black and green teas was found to be much lower than reported by other authors. Very high content of manganese in two samples of black teas from Kenya was observed. Tea is a major dietary source of manganese while the intake of other elements is negligible. In three samples, content of cadmium was found to be higher than allowed by regulations of the Health Minister of Poland.
